# SLEEP DISORDERS AND RISK OF MID- TO LATE-LIFE SUICIDE ATTEMPT

**DOI:** 10.1093/geroni/igad104.0673

**Published:** 2023-12-21

**Authors:** Amy Byers, Emily Dolsen, Yixia Li, Carolyn Gibson, Aoife O’Donovan, Thomas Neylan, Randall Kuffel

**Affiliations:** UCSF / San Francisco VA Health Care System, San Francisco, California, United States; Oakland Behavioral Health Clinic, Oakland, California, United States; NCIRE, San Francisco, California, United States; San Francisco VA Health Care System, San Francisco, California, United States; University of California, San Francisco, San Francisco, California, United States; University of California, San Francisco, San Francisco, California, United States; San Francisco VA Health Care System, San Francisco, California, United States

## Abstract

Sleep is a compelling target for suicide prevention in later life, as 50% of older adults experience sleep difficulties. The primary objective of the current study was to evaluate whether sleep disorders increase risk of late-life suicide attempt and death by suicide. We examined a nationally based cohort of over 5 million US veterans 50 years and older, who used Department of Veterans Affairs (VA) health services. Veterans with a sleep diagnosis (i.e., sleep apnea, insomnia, hypersomnia, and others) at baseline (October 1, 2011, to September 30, 2013) or earlier (October 1, 2007, to September 30, 2011) were propensity matched 1:1 with patients without these diagnoses based on demographic characteristics and Charlson Comorbidity Index (Total N=1,990,362; 995,181 each group). Secondary analyses examined psychiatric disorders as potential moderators. Fully adjusted models (additionally adjusted for psychiatric disorders) demonstrated that patients with sleep disorders had a 23% increased risk of any suicide attempt (HR 1.23, 95% CI [1.20-1.27]), while risk mitigated for lethality of attempt (HR 0.98, 95% CI [0.92-1.03]). Depression, PTSD, and generalized anxiety disorder were all approximately twice as prevalent among the sleep disorder group. Among veterans with a psychiatric disorder, any sleep disorder diagnosis was associated with a 28% increased risk for suicide attempt, which attenuated among those without a psychiatric diagnosis. In conclusion, sleep disorder diagnoses conferred increased risk for suicide attempt, especially in older adults with psychiatric disorders. These findings suggest that treatment of sleep disorders may mitigate risk of suicide attempts in older vulnerable veterans.

